# Supplementation of Saponins from Leaves of *Panax quinquefolius* Mitigates Cisplatin-Evoked Cardiotoxicity via Inhibiting Oxidative Stress-Associated Inflammation and Apoptosis in Mice

**DOI:** 10.3390/antiox8090347

**Published:** 2019-09-01

**Authors:** Jing-Jing Xing, Jin-Gang Hou, Ying Liu, Ruo-Bing Zhang, Shuang Jiang, Shen Ren, Ying-Ping Wang, Qiong Shen, Wei Li, Xin-Dian Li, Zi Wang

**Affiliations:** 1 College of Chinese Medicinal Materials, Jilin Agricultural University, Changchun 130118, China; 2 National & Local Joint Engineering Research Center for Ginseng Breeding and Development, Changchun 130118, China; 3 Intelligent Synthetic Biology Center, Daejeon 34141, Korea; 4 College of Life Science, Kyung Hee University, Seoul 446-701, Korea

**Keywords:** ginsenosides, cisplatin, cardiotoxicity, PI3K/Akt/GSK-3β, oxidative stress, inflammation, apoptosis

## Abstract

Background: Although kidney injury caused by cisplatin has attracted much attention, cisplatin-induced cardiotoxicity is elusive. Our previous studies have confirmed that saponins (ginsenosides) from *Panax quinquefolius* can effectively reduce acute renal injuries. Our current study aimed to identify the potential effects of saponins from leaves of *P. quinquefolius* (PQS) on cisplatin-evoked cardiotoxicity. Methods: Mice were intragastrically with PQS at the doses of 125 and 250 mg/kg daily for 15 days. The mice in cisplatin group and PQS + cisplatin groups received four times intraperitoneal injections of cisplatin (3 mg/kg) two days at a time from the 7th day, respectively. All mice were killed at 48 h following final cisplatin injection. Body weights, blood and organic samples were collected immediately. Results: Our results showed that cisplatin-challenged mice experienced a remarkable cardiac damage with obvious histopathological changes and elevation of lactate dehydrogenase (LDH), creatine kinase (CK), creatine kinase isoenzyme MB (CK-MB) and cardiac troponin T (cTnT) concentrations and viabilities in serum. Cisplatin also impaired antioxidative defense system in heart tissues manifested by a remarkable reduction in reduced glutathione (GSH) content and superoxide dismutase (SOD) activity, demonstrating the overproduction of reactive oxygen species (ROS) and oxidative stress. Interestingly, PQS (125 and 250 mg/kg) can attenuate cisplatin-evoked changes in the above-mentioned parameters. Additionally, PQS administration significantly alleviated the oxidation resulted from inflammatory responses and apoptosis in cardiac tissues via inhibition of overexpressions of TNF-α, IL-1β, Bax, and Bad as well as the caspase family members like caspase-3, and 8, respectively. Conclusion: Findings from our present research clearly indicated that PQS exerted significant effects on cisplatin-induced cardiotoxicity in part by inhibition of the NF-κB activity and regulation of PI3K/Akt/apoptosis mediated signaling pathways.

## 1. Introduction

Cisplatin is considered as one of the most potent chemotherapeutic agent against a verity of tumors [1]. However, its effectiveness can be often limited by tissues toxicity such as nephrotoxicity and ototoxicity, which are reported previously [2]. Currently, other factors like acute and cumulative cardiovascular complications are also been reported, which can impair the quality of patient’s life [3]. Electrocardiographic changes, myocarditis as well as cardiomyopathy are considered as its major clinical symptoms [4]. These cardiac changes leading to the max-dose reduction of cisplatin, moreover, it will also necessitate the discontinuation of chemotherapy employment [5]. Although we have not formed a comprehensive understanding on cardiotoxicity induced by cisplatin, recent researches have demonstrated that oxidative stress, apoptosis, and inflammation are commonly involved in the occurrence of cisplatin-induced injury [6]. Generally, cisplatin induces mitochondrial dysfunction [7] and decrease of antioxidants in tissues of cancer patients during cisplatin therapy [8], which lead to the overproduction of ROS and subsequent oxidative stress. Importantly, overwhelmed oxidative stress causes changes in the heart after several injections of cisplatin, like fibrosis and edema [9]. Furthermore, excessive ROS production can generate inflammation reactions through NF-κB signal pathway activation resulting in increased expression and secretion of proinflammatory cytokines in cisplatin-induced pathologies [10]. Bcl-2 plays an indispensable part in the process of cardiomyocytes apoptosis, while Bax is a main regulator of Bcl-2 activity [11]. When cisplatin induces generation of ROS, Bax is activated and transported to the mitochondrial outer membrane and changes its permeability, resulting the opening of the mitochondrial permeability transition pores (MPTPs) and the release of cytochrome C into the cytosol, and therefore causing activation of caspase 9 and its downstream caspases-dependent manner [12].

Overproduction of proinflammatory factors, injuries of immune cells, as well as turbulence of the PI3K/Akt signaling pathway, activate apoptosis altogether. Previous studies have confirmed that cisplatin induced irreversible renal dysfunctions owing to excessive cell death, which can be reduced through regulating of PI3K/Akt/GSK-3β signaling pathways. Cisplatin has been shown to modulate PI3K/Akt signal pathway to induce apoptosis in a variety of tissues [13], but its mechanism of action on cardiomyocytes remains unclear. In our study, we also testified that PI3K/Akt signaling pathway is closely related to the effect of cardioprotective effects. The activity of apoptosis-related protein kinase like caspase family members, and Bax can be stimulated by GSK-3β, finally causes apoptosis [14]. Moreover, GSK-3β can be mediated by PI3K/Akt signal pathway in a mouse model and it can also be considered as an indispensable part in the occurrence of Akt [15]. Previously, researches have confirmed that PI3K/Akt plays a vital role in the evolution of myocardial infarction as well as diabetic cardiac injuries [16]. Moreover, the up-regulation of PI3K/Akt pathway attenuates myocardial damages induced by doxorubicin [17]. GSK3β has been shown to play a defensive role against oxidation and toxicological stress through elevation of antioxidant and detoxifying enzyme levels [18].

NF-κB, which is considered response factor in an early stage, exerts significant effects in stimulating generations of various proinflammatory factors [19]. In the meantime, NF-κB combines with inhibiting NF-κB proteins (IκBs) to form a trimmer that is retained in the cytoplasm. Once IκBs are phosphorylated and degraded, NF-κB moves from the cytosol to the nucleus to regulate its target genes [20,21].

The roots of *Panax quinquefolius*, named American ginseng, has been recognized widely herb of genus *Panax* in the US and Canada, its roots and rhizomes have been employed extensively for more than 300 years in China [19]. Like the roots, the leaves of *P. quinquefolius* was rich in saponins including ginsenosides Rb1, Rb2, Rc, Rb3, Rd, Rg1, and Re. Previous studies have focused more on pharmacological activities of several saponins, which are extracted from leaves of *P. quinquefolius* (PQS), including kidney protection [22], anti-inflammation [23], anti-oxidation [24], hypoglycemic effect, etc. 

A recent report from our group has confirmed that PQS exerted significant reno-protective effects on cisplatin-evoked renal damages in mice through suppression of oxidative stress, inflammation and apoptosis [19]. Considering PQS’s better activity on cisplatin-resulted nephrotoxicity, it will be of great significance to study the protective potential of PQS on cisplatin-caused cardiac toxicities. According to the above works, from our present investigations, we supposed that PQS may have protecting potential on cardiotoxicity in a mouse model. Interestingly, we have confirmed the cardioprotective effect of PQS in cisplatin-treated mice. 

## 2. Materials and Methods 

### 2.1. Chemicals and Reagents

All standards were at least 95% pure, as confirmed by HPLC. HPLC-grade acetonitrile and methanol were purchased from Merck (Darmstadt, Germany). Cisplatin (purity ≥ 99%), was supplied from Sigma Chemicals (St. Louis, MO, USA). Hematoxylin and eosin (H&E), malondialdehyde (MDA), glutathione (GSH), superoxide dismutase (SOD), lactic dehydrogenase (LDH), and myeloperoxidase (MPO) commercial assay kits were obtained from Nanjing Jiancheng Bioengineering Research Institute (Nanjing, China). The primary rabbit monoclonal antibodies including caspase-3, cleaved caspase-3, caspase-8, cleaved caspase-8, caspase-9, cleaved caspase-9, Bax, Bcl-2, β-actin, and secondary rabbit antibodies were purchased from Cell Signaling Technology (Danvers, MA, USA) or DBOSTER Bio-Engineer Co., Ltd. (Wuhan, China). TUNEL apoptosis detection kits were provided with Roche Applied Science (No. 11684817910). Hoechst 33258 dye kits were obtained from Shanghai Beyotime Co, Ltd. (Shanghai, China). DyLight 488-labeled and SABC-Cy3 secondary antibodies were provided by BOSTER Bio-Engineer Co., Ltd. (Wuhan, China). TNF-α, IL-1β, CK, CK-MB, and cTnT commercial ELISA kits were all provided by R&D systems (Minneapolis, MN, USA).

### 2.2. Animal and Experiments Design

ICR mice (Eight-week-old, male), weighting 25~30 g, provided by Changchun YISI Experimental Animals Co., Ltd. (Changchun, China). The mice were given a standard laboratory diet and water *ad libitum* and maintained at 12 h light/dark cycle at constant temperature (23 ± 2 °C). All experimental animals’ processing project were strictly performed according to the Guide for the Care and Use of Laboratory Animals (2016). Animal experiments conducted in line with experimental protocols, and have been acknowledged and confirmed by Jilin Agricultural University Ethical Committee (Permit No.: ECLA-JLAU-18090). The selected 10 mice were randomly took in a group, 5 groups in total, and raised for two weeks before the start of formal experiment, Group 1: normal group, Group 2: cisplatin group (3 mg/kg), Group 3: PQS groups (250 mg/kg), Group 4 and Group 5: cisplatin + 125 or 250 mg/kg PQS groups, respectively. PQS was dissolved in 0.05% carboxymethylcellulose sodium in advance. Mice in group 2, 4 and 5 received four times intraperitoneal injection of cisplatin with 3 mg/kg (body weight) on the 7th, 9th, 11th, and 13th day, and mice in group 4 and 5 were administered with PQS at different doses (125 and 250 mg/kg) for 15 days. Mice in group 3 were administrated with PQS (250 mg/kg) only. Then, all mice were killed at 48 h after final injection of cisplatin. Body weights, blood and tissue samples collections were handled immediately for different purpose. Five hearts in each groups were swiftly and carefully been put into liquid nitrogen, while other hearts were fixed in formalin. Heart serum sample collections were also been promptly segregated by refrigerated centrifuge for the following analysis.

### 2.3. Biochemical Parameters Determination 

#### 2.3.1. Cardiac Biomarkers 

Activities of serum cardiac enzymes CK (Cat. No. MM-58997), CK-MB (Cat. No. MM-0839M1), CK-MB (Cat. No. MM-43703M1), and cTnT (Cat. No. MM-0945M1) were measured by using ELISA kits according to the commercial protocols. 

#### 2.3.2. Assessment of Cardiac Oxidative Stress

Heart homogenates were used to estimate different oxidative stress parameters. The heart tissues were homogenated in 50 mM phosphate buffer (pH 7.4). The resulting suspension was then centrifuged at 3000× *g* for 10 min twice at 4 °C, and the supernatant was used for the detection of GSH, MDA and SOD. In brief, the levels of oxidative indexes in heart homogenates were detected by commercial kits.

#### 2.3.3. Assessment of Proinflammatory Cytokine 

MPO was determined via tissues homogenate. In order to measure the MPO activity, we also measured the rate of oxidation of odianisidine and the absorbance was 460 nm, the MPO activity was calculated and expressed by U/mg protein. Moreover, serum TNF-α and IL-1β were assayed by mouse TNF-α and IL-1β reading ELISA plates at a wavelength of 450 nm.

### 2.4. H&E Staining 

The hearts sections from the normal as well as cisplatin groups were disposed with paraffin 10% buffered formalin. Sections were cut into approximately 5 μm thickness and were stained with H&E staining observe and identify sections histology by light microscope (Leica TCS SP8, Leica Microsystems, Mannheim, Germany) [20].

### 2.5. Immunohistochemistry 

Briefly, the sections were deparaffinized and rehydrated with xylene and various concentrations of alcohol solutions [21]. TBS was used to wash all sections, then they were incubated with 1% BSA for 2 h. Then, they were washed, and were incubated at 4 °C for 12 h with primary antibodies including mouse polyclonal anti-Bax (1:200) and anti-Bcl-2 (1:200), followed by mouse and rabbit secondary antibodies for 1 h. Substratum was added to the tissues for 1 h after DAB staining. The positive staining was detected majorly by brown color in the cytoplasm or nucleus of the positive cells. A light microscopy (Leica, DN750, Berlin Germany) was also used to observe and record the changes.

### 2.6. Immunofluorescence and Hoechst 33258 Staining

We used primary antibodies like COX-2 (1:200) and iNOS (1:200) in 4 °C overnight, and then all selected sections were exposed to Dylight448-labeled secondary antibody. DAPI staining was used to visualize nucleus followed by PBS washing. Light microscope (LEICA DM 2500, Berlin, Germany) was used to observe their changes. Hoechst 33258 was conducted as mentioned earlier with slight modifications. Briefly, the heart tissues were removed out and sealed in 10% formalin solution. After randomly chose three tissues from every group. Three other samples were chopped into 5 μm sections and dyed by specific stains (10 μg/mL). And then, we used PBS to wash all the sections for three times, fluorescence microscope was used to observe stained nuclei. We also used Image-Pro plus 6.0 to quantify the staining results.

### 2.7. Western Blotting

Radio Immunoprecipitation Assay (RIPA) buffer was used to split proteins. We prepared 12% SDS polyacrylamide gels and transferred the proteins (50 μg/lane) to a polyvinylidene difluoride (PVDF) membrane. 5% non-fat milk insulted with Tris-buffered saline (TBS) which was made up of 0.1% Tween-20 for more than 2 h at room temperature, then PBS was used to wash the PVDF membrane three times before incubating in primary antibodies at 4 °C for 12 h. Thereafter, the membrane was shacked for half an hour at room temperature before being washed three times by TBST, and 8 min for each time. Latterly, secondary mouse and rabbit antibodies was separately incubated the membrane for 2 h. Eventually, Emitter Coupled Logic (ECL) substrate (Pierce Chemical Co., Rockford, IL, USA), which preserved in 4 °C was 1:1 mixed to detect the expressions of all proteins. We also used Image plus 6.0 software (Media Cybernetics, Rockville, MD, USA) to analyze date.

### 2.8. Statistical Analysis

All data referenced were expressed as the mean ± S.D. and analyzed with SPSS 19.0 (SPSS, Chicago, IL, USA). Differences among experimental groups were conducted by one-way of variance (ANOVA). Statistical significance was defined as *p* < 0.05 or *p* <0.01.

## 3. Results

### 3.1. Typical HPLC Chromatogram of PQS

**Figure 1:**
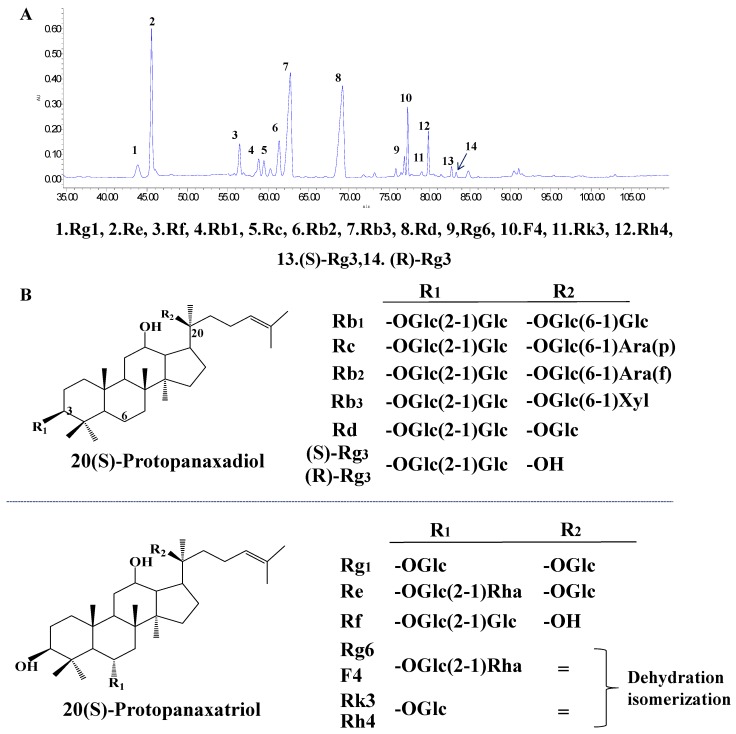
(**A**) Fourteen saponins from leaves of *P. quinquefolius* are confirmed by HPLC analysis, chromatograms and chemical structures of these saponins mainly includes panaxadiol-type ginsenosides Rb1, Rc, Rb2, Rb3; Rd, 20(S)-ginsenoside Rg3, 20(R)-ginsenoside Rg3 and panaxatriol-type ginsenosides Rg1, Re, Rf, Rg6, F4, Rk3, and Rh4. (**B**) The structures of these fourteen saponins.

High performance liquid chromatography (HPLC) was used to determine and identify the components of PQS. We authenticate all compositions like Rg1, Re, Rf, Rb1, Rc, Rb2, Rb3; Rd, Rg6, F4, Rk3, Rh4, (S)-Rg3, and (R)-Rg3 via comparing the retention times in mixed saponins standard. The chromatograms and structures are concluded in Figure 1. 

### 3.2. PQS Protects Against Cisplatin-Induced Cardiotoxicity

**Figure 2:**
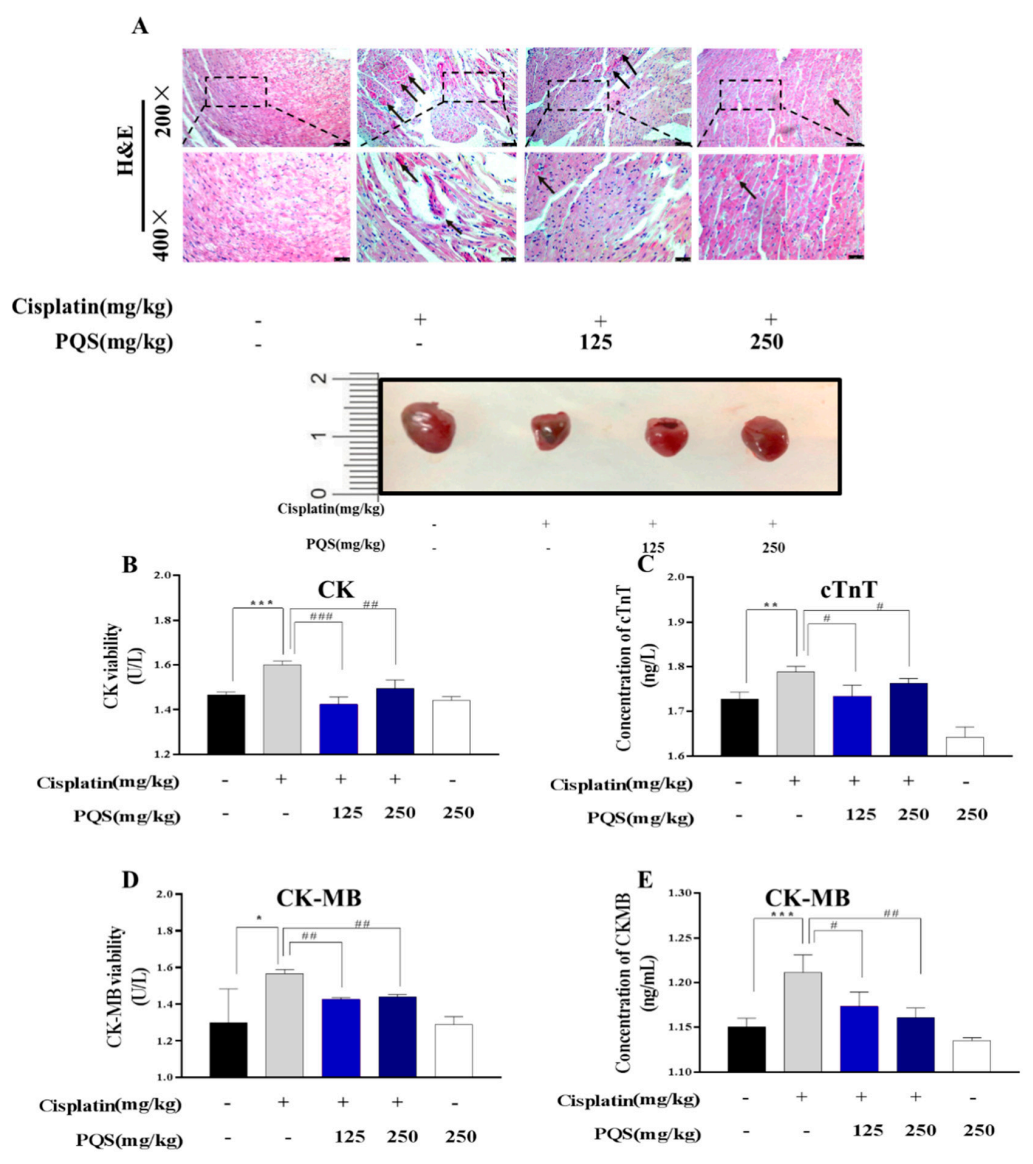
Effect of PQS on cisplatin-induced changes in heart tissues of mice (**A**). Cisplatin + PQS (125 mg/kg), Cisplatin + PQS (250 mg/kg) groups (H&E × 200). Effects of cisplatin and PQS on the serum levels of related markers CK (**B**), cTnT (**C**), viability of CK-MB (**D**), and concentration of CK-MB (**E**). All data were expressed as mean ± S.D. * *p* < 0.05 or ** *p* < 0.01 or *** *p* < 0.01 comparing with normal group. *^#^ p* < 0.05 or *^##^ p* < 0.01 or *^###^ p* < 0.01 comparing with cisplatin group.

Figure 2 showed that administration of cisplatin injection (5 mg/kg) for 4 times leaded to elevation of CK, CK-MB competence as well as cTnT level comparing with normal group. These changes indicated that cardiac injury can be induced by cisplatin *in vivo*. However, PQS resulted in reduction (*p* < 0.05) in the above-mentioned indicators. Furthermore, histologic sections from the normal group shows that cardiac muscle fibers is regular, however, in the cisplatin-injected group, abundant degeneration in cardiac muscle fibers can be noticed (Figure 2B, E). Mice pretreated with PQS indicated similar forms, which showed that PQS (250 mg/kg body weight) was exerting no impairments on heart

### 3.3. PQS Inhibits Oxidative Stress Induced by Cisplatin Treatment 

**Figure 3:**
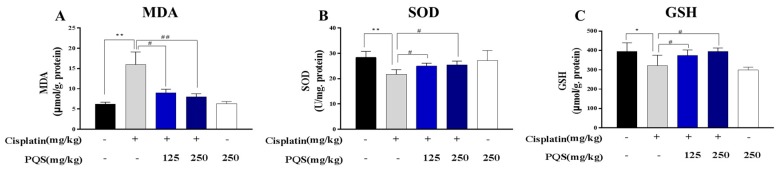
Effects of PQS on the levels of (**A**) MDA, (**B**) superoxide dismutase (SOD) and (**C**) glutathione (GSH). All data were expressed as mean ± S.D. * *p* < 0.05 or ** *p* < 0.01 comparing with normal group. *^#^ p* < 0.05 or *^##^ p* < 0.01 comparing with cisplatin group.

To assess the cardiac markers of oxidative stress injury, GSH and SOD level in heart tissues were tested. As shown in Figure 3, PQS attenuated significantly the decline of myocardial SOD induced by cisplatin (*p* < 0.05) compared to the normal group. GSH content was significantly reduced by cisplatin, compared with the normal group (*p* < 0.05), which were ameliorated by PQS administration evidently (*p* < 0.05). MDA is an important parameter reflecting the potential antioxidant capacity of the body, which can reflect the lipid peroxidation rate and intensity of the body, and can also indirectly reflect the degree of tissue peroxidation damage. The level of MDA was increased after injections of cisplatin, however, a significant decrease of MDA level was observed after treatment with PQS. These data clearly demonstrated that PQS alleviated cisplatin-caused cardiac oxidative stress injuries (Figure 3A–C).

### 3.4. Effect of PQS on Cardiac Inflammation 

**Figure 4:**
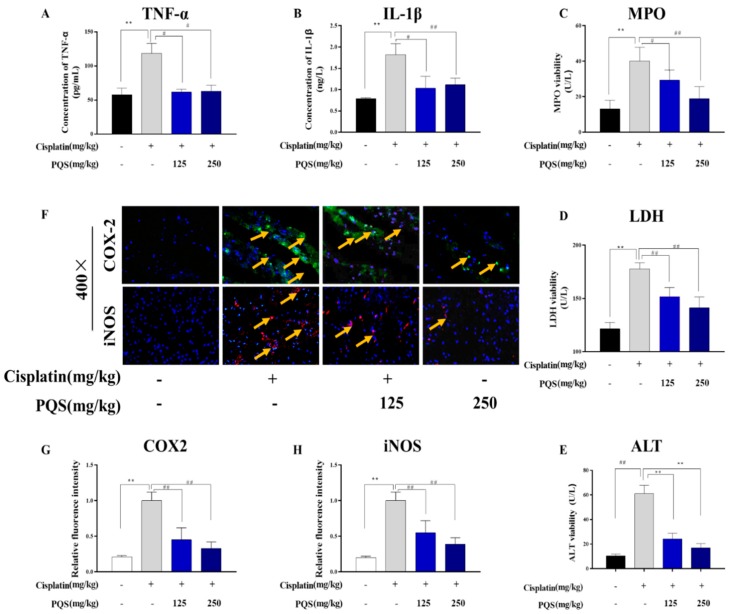
Effect of PQS on cisplatin-induced changes in inflammatory markers in heart tissues of mice. (**A**) Tumor necrosis factor-a (TNF-α); (**B**) Interleukin-1β (IL-1β); (**C**) Myeloperoxidase (MPO) activity; (**D**) Lactate dehydrogenase (LDH); (**E**) ALT activity; (**F**) PQS exerted great changes on expression of COX-2 and iNOS in heart tissues, the expression levels of COX2 (Green) and iNOS (Red) in tissue section isolated from different groups were assessed by immunofluorescence. (**G**) Quantitative analysis of scanning densitometry for cleaved COX-2 (**H**) Quantitative analysis of scanning densitometry for cleaved iNOS. All data were expressed as mean ± S.D. ** *p* < 0.01 comparing with normal group. *^#^ p* < 0.05 or *^##^ p* < 0.01 comparing with cisplatin group.

In order to better understand the anti-inflammatory effects of PQS, levels of TNF-α and IL-1β in serum, and activities of LDH and MPO as markers for reflecting neutrophil infiltration were detected. Serum levels of TNF-α and IL-1β showed remarkable elevation for nearly more than 2-folds in mice treated with cisplatin only, and near 1-fold on mice treated with PQS comparing to normal group (*p* < 0.001). Likewise, MPO activity in heart tissues were higher in cisplatin group than that in normal group. Co-administration of PQS significantly abolished the MPO activity. A significant rise in LDH activity illustrated the impairment of heart induced by cisplatin challenging, and PQS significantly decreased these serum-marker enzymes (Figure 4A,C,D,E) (*p* < 0.01). Moreover, pro-inflammatory COX-2 and iNOS levels in the heart were assessed through immunofluorescence. As evidence from immunofluorescence staining, COX-2 and iNOS levels were elevated in cisplatin group. However, mice receiving PQS lowered the expressions than the mice treated with cisplatin alone, such alterations were significantly inhibited (Figure 4B,F,G). 

### 3.5. Effects of PQS Treatment on Cisplatin-Induced Inflammatory Markers 

**Figure 5:**
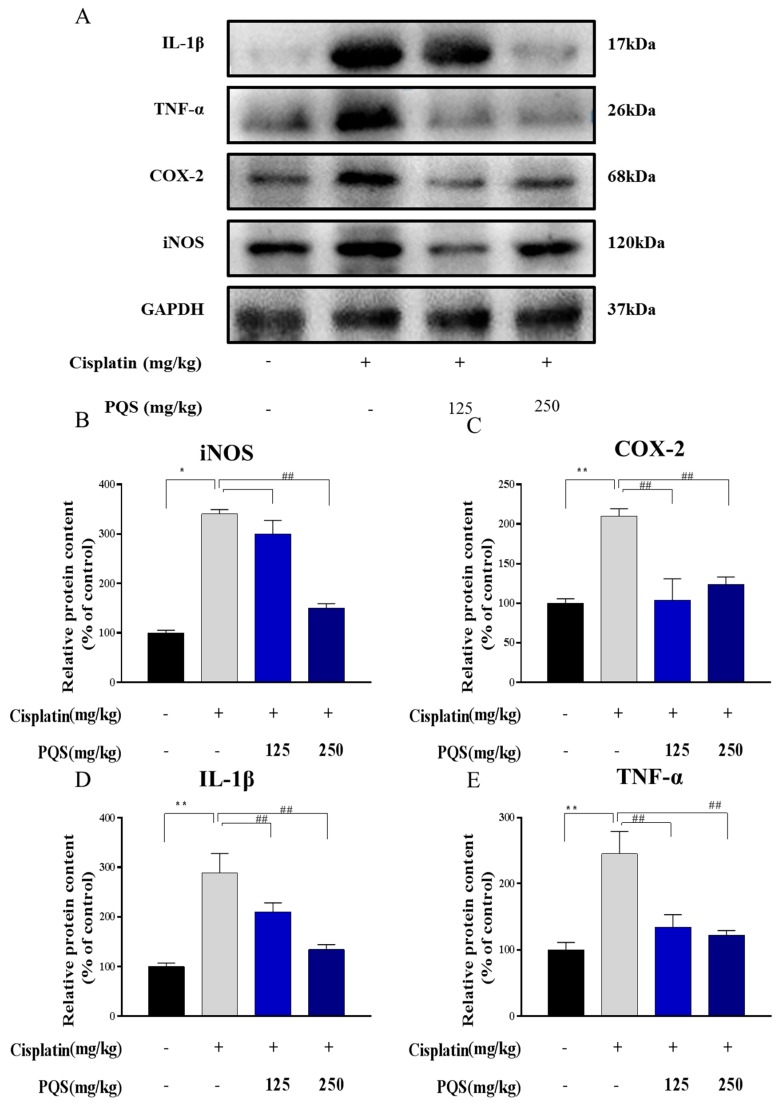
The expression of iNOS, COX-2, TNF-α and IL-1β were measured by western blotting analysis (**A**). Quantitative analysis of scanning densitometry for iNOS (**B**); COX-2 (**C**); IL-1β (**D**); TNF-α (**E**). All data were expressed as mean ± S.D. * *p* < 0.05 or ** *p* < 0.01 comparing with normal group. *^##^ p* < 0.01 comparing with cisplatin group.

Additionally, reduction of overproduction of iNOS and COX-2 in the heart tissues by the PQS pretreatment were confirmed by western blotting analysis (Figure 5) (*p* < 0.05, *p* < 0.01). The expression levels of TNF-α and IL-1β in the heart tissues were also determined by western blotting. As expected, expression levels of proinflammatory factors like TNF-α and IL-1β induced by cisplatin induced by 2.37- and 2.44-fold respectively, when compared to the normal group (Figure 5) (*p* < 0.05 or *p* < 0.01) comparing with normal group.

### 3.6. Effects of PQS on the NF-κB Signaling Pathway

**Figure 6:**
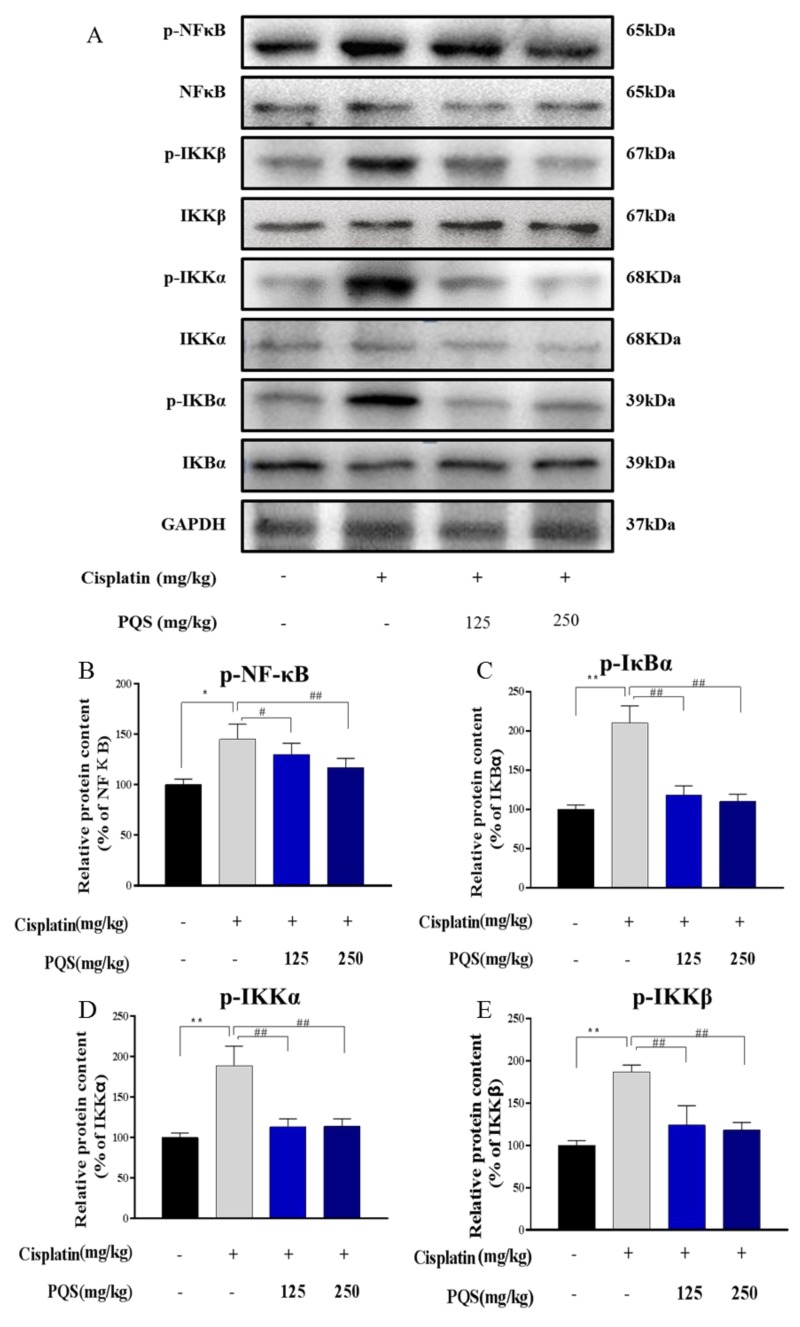
(**A**). Quantitative analysis of scanning densitometry for p-NF-κB (**B**); p-IκBα (**C**); p-IKKα (**D**); p-IKKβ (**E**). All data were expressed as mean ± S.D. * *p* < 0.05 or ** *p* < 0.01 comparing with normal group. *^#^ p* < 0.05 or *^##^ p* < 0.01 comparing with cisplatin group.

To determine whether PQS can improve cisplatin-induced cardiotoxicity by reducing cisplatin caused inflammation. The effects of PQS pretreatment on cisplatin-activated NF-κB signal pathway were tested by western blotting analysis in this study. Treatment with PQS (125 and 250 mg/kg) decreased the levels of p-IKKα/β, p-IκBs and p-NF-κB, which demonstrated that PQS can effectively recede inflammation evoked by cisplatin (Figure 6A–E).

### 3.7. PQS Attenuates the Intrinsic Mitochondrial Apoptotic Pathway In Vivo

**Figure 7:**
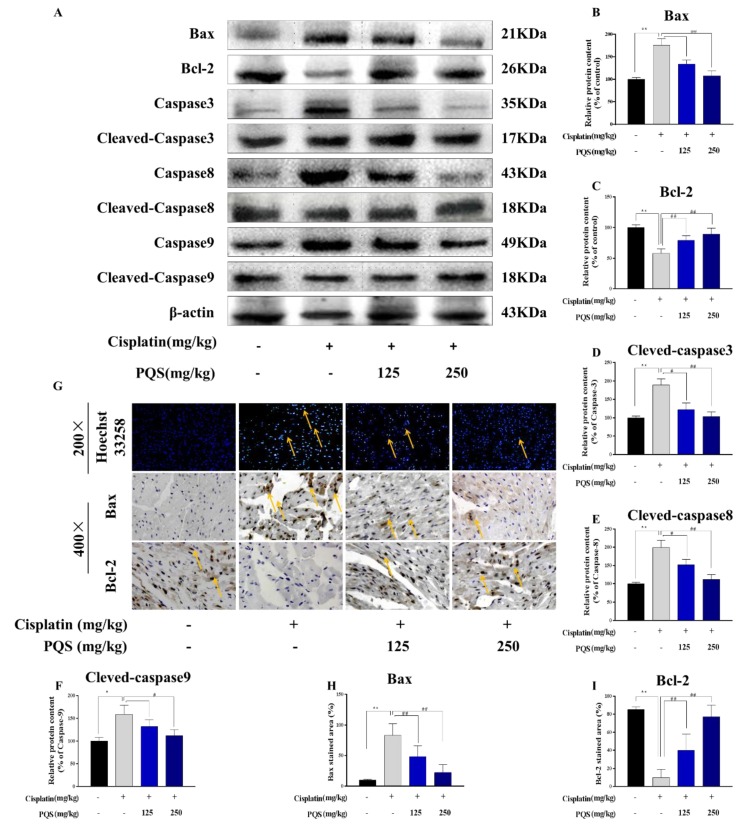
(**A**) The blots of Bax, Bcl-2, Bad, caspase 3, cleaved caspase 3, caspase 8, cleaved caspase 8 and caspase 9, cleaved caspase 9 were standardized to that of β-actin; Quantitative analysis of scanning densitometry for Bax (**B**), Bcl-2 (**C**), Caspase-3 (**D**), Caspase-8 (**E**), Caspase-9 (**F**). (**G**) Representative photomicrographs of cardiac immunohistochemically staining for Hoechst 33258, (**H**) Bax staining area. The percentage of apoptosis (**I**) and Bcl-2 staining area in indicated groups. Scale bars data were expressed as mean ± S.D. * *p* < 0.05 or ** *p* < 0.01 comparing with normal group. *^#^ p* < 0.05 or *^##^ p* < 0.01 comparing with cisplatin group.

In order to explore the underlying mechanism of the reduction in apoptosis in PQS treatment, we evaluated Bcl-2 and Bax by western blotting (Figure 7B). Mice subjected to cisplatin, the ratio of Bcl-2 and Bax was reduced in comparison to the normal group (*p* < 0.05). Activation of Bax and decrease of Bcl-2 (Figure 7E) were confirmed by immunohistochemical analysis. Treatment with PQS (125 and 250 mg/kg) significantly reversed Bax, Bad, caspase-3, caspase-8, and caspase-9, however, elevated the level of Bcl-2. All data supported that PQS can inhibit apoptotic pathway activation. Interestingly, treatment with PQS led to an increase in this ratio to 1.3 for Bcl-2, and decreased this ratio by 42.7% for Bax, these findings indicated that PQS might block apoptosis through a mitochondrial pathway mediated by the relative ratio of expression of Bcl-2 and Bax in the mitochondria. In this study, apoptosis was further verified by Hoechst 33258 staining in heart tissues. As shown in Figure 7E, mice injected with cisplatin alone showed significantly increased positive staining cells with condensed nuclei. However, comparing with cisplatin group, less apoptotic cells were observed in the cardiac sections in PQS pretreatment group.

### 3.8. PQS Attenuates Cisplatin-Induced through PI3K/ Akt/ GSK-3β Signal Pathway

**Figure 8:**
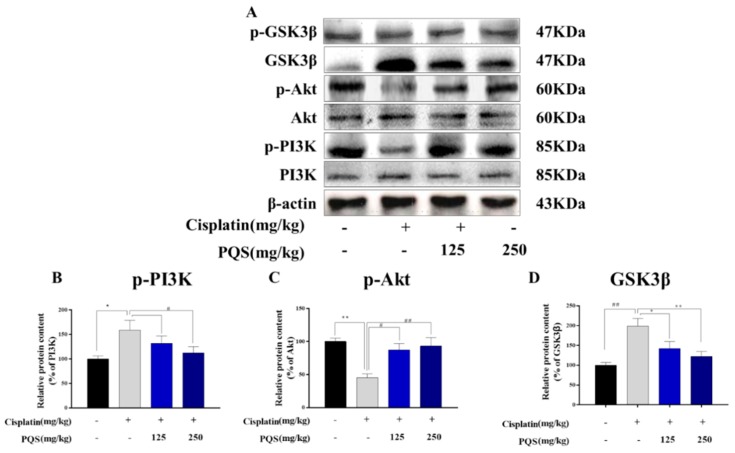
Mice pretreated with PQS or saline for 7days, given with or without cisplatin at day 7th, 9th, 11th and 13th. (**A**) Representative western blots depicting total and phosphorylated PI3K, Akt, GSK-3β. Quantitative analyses of the p-PI3K/PI3K, p-Akt/Akt, and p-GSK-3β/GSK-3β expression ratios are shown (**B**–**D**). All data were expressed as mean ± S.D. * *p* < 0.05, ** *p* < 0.01 comparing with normal group. *^#^ p* < 0.05, *^##^ p* < 0.01 comparing with cisplatin group.

Mice pretreated with PQS or saline for 7 days, given with or without cisplatin at day 7th, 9th, 11th and 13th. (Figure 8A) Representative western blots depicting total and phosphorylated PI3K, Akt, GSK-3β. Quantitative analyses of the p-PI3K/PI3K, p-Akt/Akt, and p-GSK-3β/GSK-3β expression ratios are shown (Figure 8B–D). 

### 3.9. Mechanism of PQS Improving Cardiac Toxicity Induced by Cisplatin 

**Figure 9:**
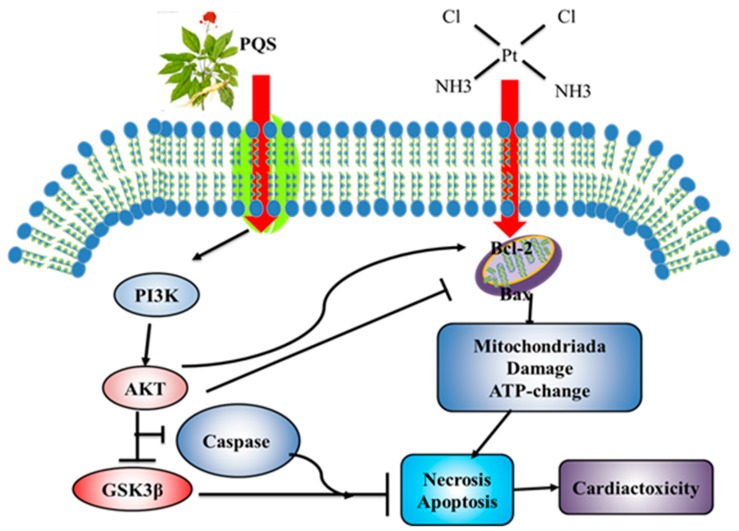
Scheme Summarizing the Inhibition of Cisplatin-Induced Cardiotoxicity by PQS via the Upregulation of PI3K/Akt/GSK-3β Mediated Inhibition of Oxidative Stress Inflammation and Apoptosis.

When cisplatin enters the body, it promotes the expression of Bax and inhibits the increase of Bcl-2, which leads to mitochondrial dysfunction and disorder of ATP synthesis, leading to cell apoptosis and necrosis, and finally cardiac toxicity. However, PQS improve cisplatin-induced cardiac toxicity through PI3K/Akt /GSK-3β pathway and caspase family protein expressions as described in Figure 9.

### 3.10. PQS Attenuates Cisplatin-Induced through PI3K/Akt/GSK-3β Signal Pathway

**Figure 10:**
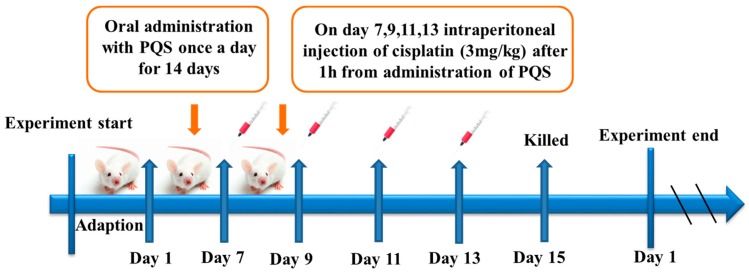
Experimental Flow Chart.

Mice pretreated with PQS or saline for 7 days, given with or without cisplatin at day 7th, 9th, 11th and 13th. Mice were dissected at 15^th^ day and serum and tissue samples were collected for future detection as described in Figure 10.

## 4. Discussion

Cisplatin is acknowledged as one of the widely employed chemotherapy agents to treat solid tumors in clinic. Nevertheless, its toxicity can also limit its usages, moreover higher doses of cisplatin also induced serious cardiotoxicity. However, its major and concrete mechanism of cardiotoxicity resulted by cisplatin is poorly understood. Some evidence has previously confirmed that oxidative stress, inflammation and apoptosis plays an important part in cardiotoxicity evoked by cisplatin [25]. 

The total saponins (ginsenosides, PQS) from leaves are accessible to extract and isolate [26]. Previous studies have confirmed that it had beneficially effective effects on treating coronary heart disease. Moreover, researches have confirmed that it has been extensively used in acute myocardial infarction clinically. From the results of our current works, we have investigated its effective effects on cardiac protection. Moreover, our current researches have also provided its medicinal value and exploited more market for PQS to reduce patients’ side effects induced by cisplatin on cardiac injury in clinical research.

Cisplatin can result in over-generation of ROS, which will alter the cells’ structures, functions and integrity. CTnT, which is acknowledged as a specific marker of cardiac dysfunction, because it can be released into the body after chemotherapeutic treatments. Moreover, myocardial damages can be resulted by overproduction of cTnT [27]. Chemotherapy diminishes the normal homeostasis of the body, which is particularly applicable for cisplatin treatment. Previous studies have reported that cisplatin exposure usually results in cardiotoxicity, which could be a secondary event following increased lipid peroxidation of cardiac membranes that results in irreversible modification of membrane structures and functions with the consequent leakage of cardiac enzymes as well as cTnT [20]. In line with previous study, our results showed that the cTnT concentration and some cardiotoxicity-associated markers, for example, LDH, CK and CK-MB were increased after cisplatin challenge when compared to the normal group. Furthermore, we also found that protective potentials of PQS in myocardial tissue were shown evidently in histopathological examinations. Mice administered with cisplatin alone showed histopathological changes in myocardial tissues. However, PQS plus cisplatin can alleviate the above-mentioned changes. We also found that normal myocardial morphology structure can be observed in mice treated with PQS (250 mg/kg) alone. 

The decreasing levels in GSH levels and decreased viabilities of SOD enzyme can be also marks of oxidative stress caused by cisplatin. All radicals can result massive damages in various tissues, Oxidative stress and inflammation are closely interrelated in the biological systems and this interaction facilitates the progression of cardiotoxicity induced by cisplatin [21]. ROS has not only direct deleterious effects on bodies, but induces the activation of the redox sensitive NF-κB when the concentration of ROS is high, then causing the high levels of the proinflammatory cytokines, such as TNF-α and IL-1β which play a vital role in some inflammation-associated signaling molecules [22]. Therefore, it is also reasonable to conclude that cardiac toxicities resulted by challenging of cisplatin maybe partly due to the activation of inflammation response. Some studies have also confirmed that inflammation can also promote the lipolysis [23], which can be a persuasive explanation on weight loss caused by cisplatin. Moreover, as one of the leading genes for NF-κB, COX-2 is placed at the center of a myriad of mechanisms on tissue injuries during the produce of vasoactive and pro-inflammatory responses [24]. Augmented oxidative stress can be resulted by TNF-α and IL-1β through activating COX-2 in inflammatory reactions [25]. Admittedly, the activation of neutrophils on inflammation responses can induce physical damages via the production of many pro-inflammatory and pro-oxidative enzymes, such as MPO, GSH, SOD and MDA [26]. Our results were consistent with the reported findings previously, significant increase of TNF-α and IL-1β accompanied by elevation of COX-2 protein and MPO can be observed in the cisplatin-injected groups. When tissue cells are damaged by stimulations; ALT in the cells will be released, resulting in increased ALT value [27]. We also found that cisplatin could increase the level of ALT. Interestingly, PQS can remarkably reduce these alterations indicating its potentials on anti-inflammatory. All results are in accordance with other reports on anti-inflammatory effect of PQS, which may be attributed to the scavenging of free radicals [28,29].

During the pathogenesis cardiotoxicity, inflammation, which has been extensively acknowledged as a vital contributor [30]. The transcription of inflammatory indicators, for example, TNF-α, IL-1β, COX-2 and iNOS can be also specifically triggered via activating the NF-κB [31] pathway. Moreover, IL-1β is also play a vital role in the process of inflammation [32]. Our current researches showed that, PQS can diminish inflammation responses resulted by TNF-α and IL-1β. These results were also confirmed by reducing levels of iNOS and COX-2 levels, suggesting suppression of inducible enzymatic pathways. In conclusion, these findings provide a potential for PQS on improving cardiotoxicity associated inflammatory response. In this study, PQS suppressed NF-κB activation in cisplatin induced cardiotoxicities, evidenced by decreased expressions of p-IKKα, p-IKKβ, p-IκBα, and p-NF-κB.

Recent evidence suggested cisplatin-induced cardiotoxicity is related to apoptosis. Various substrates apoptosis-associated proteins like Bax, Bad and caspase family members can trigger the apoptotic responses [33,34]. Other proteins like Bcl-2, Bcl-XL can act as antiapoptotic markers, which may cause inhibitions in some apoptotic reactions. Previous investigations have demonstrated that PI3K/Akt is considered as a prosurvival function in cardiac tissues challenged by oxidations and apoptosis induced by various stimulations [35,36]. Once Akt signal pathway is activated, it can confer cell exist by activating its cytoplasmic targets, like its downstream proteins - GSK-3*β* as well as other apoptotic indicators like Bcl-2, Bax, and caspase-9 [37]. In this study, we showed that phosphorylated PI3K/Akt levels on cisplatin-treated mice were lower than those mice untreated with cisplatin. From our results, we demonstrated GSK3β can be significantly promoted in the cisplatin-treated mice. These results can be served to decrease cardiomyocyte apoptosis, and preserve heart functions. Moreover, our western blotting analysis indicated that elevation of PI3K/Akt signaling pathway as well as the increased expression of GSK3β can serve a cardioprotective effect in this mouse model. Our data are in line with previous studies. Furthermore, we also showed that levels of caspase family members’ proteins in the myocardium of PQS-pretreated mice were less than those of the untreated mouse. Importantly, we also found that the expression level of Bcl-2 can be reversed by pretreatment with PQS. 

## 5. Conclusions

In conclusion, our researches revealed the protective potentials of PQS against cisplatin-evoked cardiotoxicity, and its mechanism may be partly attributed to the inhibition of oxidative stress, inflammation and apoptosis via PI3K/Akt/GSK-3β signaling pathway.

## Abbreviations

The following abbreviations are used in this manuscript:

CK	Creatine kinase
MPO	Myeloperoxidase
CK-MB	Creatine kinase isoenzyme MB
CTnT	Plasma cardiac troponin T Glutathione
PQS	Panax quinquefolius saponins
IL-1β	Interleukin-1β
SOD	Superoxide dismutase
LDH	Lactate dehydrogenase
TNF-α	Tumor necrosis factor-α
H&E	Hematoxylin and Eosin
NF-κB	Nuclear factor-kappa B
Akt	Protein kinase B
PI3K	Phosphatidylinositol 3-kinase
